# The profile of people undergoing lower limb amputations at Groote Schuur Hospital

**DOI:** 10.4102/ajod.v13i0.1152

**Published:** 2024-02-14

**Authors:** Katleho Limakatso, Jenna Tucker, Lennie Banda, Cheyne Robertson, Romy Parker

**Affiliations:** 1 Department of Anaesthesia and Perioperative Medicine, Faculty of Health Sciences, University of Cape Town, Cape Town, South Africa; 2 Department of Health and Rehabilitation Sciences, Faculty of Health Sciences, University of Cape Town, Cape Town, South Africa

**Keywords:** amputation, characteristics, risk factors, hospital, Western Cape

## Abstract

**Background:**

The annual incidence of lower limb amputations (LLA) at Groote Schuur Hospital is rising gradually. However, little is known about the sociodemographic and clinical profiles of people undergoing these limb amputations.

**Objectives:**

To collect and analyse data to describe the sociodemographic, health and amputation profiles of people who have undergone LLA at Groote Schuur Hospital.

**Method:**

A descriptive retrospective chart review was conducted using a sample of 107 participants who had undergone LLA at Groote Schuur Hospital between January 2019 and July 2020. A customised assessment tool was used to extract data on the sociodemographic, health and amputation profiles of patients who had LLA. Data were analysed descriptively.

**Results:**

Sixty per cent of the patients who had undergone LLA at Groote Schuur Hospital were women. Most of the patients were over the age of 60 years and had not completed school and were pensioners or unemployed, with very low income and multiple co-morbidities including poorly controlled diabetes.

**Conclusions:**

Complications because of uncontrolled diabetes were the primary indication for LLAs at Groote Schuur Hospital. Therefore, health literacy projects are indicated to address chronic diseases of lifestyle, which, in turn, may reduce the overall burden of LLA, particularly on the South African under-resourced healthcare system.

**Contribution:**

The results of this study may help us identify key factors that predispose patients to LLAs. Consequently, this may help us identify key areas for prevention and better management of diseases that can result in complications that indicate the need for amputation.

## Introduction

Amputation is defined as the removal or loss of a body part or limb. Approximately 356 million limb amputations are conducted globally every year (Moxey et al. 2011). Of these, roughly 90% are linked to diabetes (Moxey et al. 2010; Tiwari et al. 2012). Although limb amputations are performed for several reasons including cultural practices, they are chiefly performed after severe trauma, disease or congenital complications (Khan et al. 2020). It is estimated that approximately 1200 lower limb amputations (LLAs) are conducted annually because of diabetic complications in the Western Cape province of South Africa, with people experiencing various challenges post-operatively (Dunbar et al. 2015).

A limb amputation is associated with functional disability and psychological distress (Fuchs, Flor & Bekrater-Bodmann 2018; Ennion & Yu 2019). People with amputation often suffer from depression and anxiety and experience problems with ambulation (Padovani et al. 2015). In consideration of this, it is unsurprising that people with amputations have a poor health-related quality of life and are at a higher risk of being disabled and unemployed (Tutak et al. 2020). Although severe medical conditions may be remedied by undergoing an amputation, further amputation-related problems can persist beyond the period of hospitalisation (Ciufo et al. 2019).

For example, a recent study revealed a high prevalence of depression and anxiety in African people with limb amputations (Faraj, Mutavi & Gitau 2022; Roșca et al. 2021). In addition, a systematic review evaluating the effects of amputations found significant activity limitations and participation restrictions in people with amputations (Behera & Dash 2021). These findings are consistent with those of a South Africa-based study that identified care priorities for people with LLAs following hospital discharge (Limakatso 2022). In the early stage after amputation, the participants expressed the need for help with their mental health and psychological and spiritual well-being. In the long-term, however, the participants prioritised the need for living a functional and normal life, with respect and dignity like everyone else. These care priorities are context-specific and may vary from those of people with upper limb amputations. Therefore, understanding the context and characteristics of patients with amputations is essential for providing patient-centred care.

Currently, the sociodemographic and clinical profile of people undergoing lower limb amputations at Groote Schuur Hospital is not well understood. Considering the rising incidence of amputations in South Africa, understanding the profile of people accessing the healthcare system for limb amputations has the potential to enhance care and recovery (Manickum et al. 2019). Global studies have shown that being male, over 45 years of age, having multiple co-morbidities (e.g. diabetes) or a low level of health literacy are all independently associated with an increased risk of having amputation surgery (Lin et al. 2020). Exploring these variables in the South African population may help us identify key factors that make patients vulnerable to needing LLAs. Consequently, this may help us identify key areas for prevention and better management in people with conditions in which complications may indicate the need for amputation. To this end, we conducted this study with the aim of describing the sociodemographic, health and amputation profiles of people who have undergone LLAs at Groote Schuur Hospital.

## Research methods and design

### Study design

A descriptive retrospective cross-sectional chart review was conducted. This study design is time-efficient, cost effective and scaled, with resources devoted mostly to data collection (Vassar & Matthew 2013).

### Research setting

The study was conducted at Groote Schuur Hospital – a tertiary-level healthcare institution in Cape Town, South Africa. Groote Schuur Hospital is a government-funded institution providing amputation services to people within and out of the Cape Town metropolitan area.

### Population

The population of interest is people with acquired limb amputations. The sampling frame is adults (≥18 years) who have undergone surgical or traumatic upper or lower limb amputations at Groote Schuur Hospital.

### Sample size determination

The required sample size was calculated using the Yamane Formula (Yamane 1967) $\left[n=\frac{N}{1+N(e)2}\right]$ where N is the study population, and *e* is the constant. Using a population of 167 (number of amputations at GSH per annum) and a constant of 0.05 for a 95% confidence interval, 118 participants were required.

### Procedure

Three researchers (JT, LB and CR) retrieved the names of people who had undergone limb amputations from a pre-existing and HREC-approved database held in the acute care surgical unit of Groote Schuur Hospital (acute care surgery online database: HREC 020/2018. Valid until 30 September 2021). This registry included patients who had undergone limb amputations and provided consent to be contacted or have their data used for research purposes.

The researchers randomly selected 20 names from the registry at a time and accessed the relevant folders from the Groote Schuur Hospital records department in tranches of 20 folders at a time until the patient list was exhausted, or the desired sample size was attained. The researchers extracted relevant information and entered it into the Microsoft Excel spreadsheet tool. Once data were captured, the folders were returned to the records department.

### Outcome measure

A pre-piloted customised data extraction tool was used to collect data on the sociodemographic and clinical profile of patients who had undergone amputation surgery at Groote Schuur Hospital between 2019 and 2020: demographic, health and amputation-related characteristics.

### Statistical analysis

Data were analysed using non-parametric statistics because numerical data were not normally distributed. The results are therefore reported as a median with an interquartile range (IQR). The remaining data were analysed descriptively.

### Ethical considerations

Ethical approval to conduct this study was sought and obtained from the University of Cape Town, Faculty of Health Sciences, Human Research Ethics Committee (HREC) [Ref:164/2021]. The study was conducted in accordance with the ethical principles for conducting research in humans as delineated in the Declaration of Helsinki (Cantín 2014). All patients who underwent amputation surgery and were included in this study had signed informed consent for their data to be entered into the registry. While the methodology of chart review is regarded as low risk, there remains a risk of breaking patient confidentiality. To reduce this risk, all data were de-identified at the point of collection. In addition, precautions for non-maleficence included logging patient folders in and out of patient records to prevent loss of information that may be needed for future treatment. Finally, the ethical principle of beneficence was addressed through presentation of these results to staff and students at the hospital and through this publication to increase knowledge about the profile of people who undergo amputation in this setting.

## Results

One hundred and eighty-seven people who had limb amputation between January 2019 and July 2020 were identified from the registry. The folders of 107 people were retrieved. Other patient files could not be found.

### Sociodemographic characteristics

The sociodemographic characteristics are presented in Table 1. The study included 107 participants (male [*n* = 50]; female [*n* = 57]) with a median [IQR] age of 61 [57–86]. Most participants were pensioners (47.66%) and had not completed school (*n* = 86; 80.34%), and only one lived outside the city of Cape Town. Over 52% of the participants earned <ZAR1690 per month, meaning their treatment was fully subsidised.

**TABLE 1:** Sociodemographic characteristics (*N* = 107).

Characteristic	Number of participants	%
**Sex**		
Male	50	46.73
Female	57	53.27
**Income**		
Earns <ZAR1690.00 per month (H0)	56	52.30
Earns ZAR1690.00-7000.00 per month (H1)	43	40.20
Earns > ZAR7000.00 per month (H2)	8	7.50
**Education**		
None	18	16.82
Primary (Grade 7 or less)	30	28.04
Incomplete high school (Grades 8–11)	39	36.45
Completed high school (Grade 12)	19	17.75
Special needs education	1	0.93
**Employment status**		
Employed	24	22.42
Retired (pensioner)	50	47.66
Unemployed	31	28.97
Disabled	2	1.87

### Health characteristics

The participants presented with a variety of co-morbidities (Table 2). The most prevalent of these were diabetes mellitus type II (80.25%) and hypertension (69.14%). Most participants presented with more than one comorbidity. Roughly 55% of the participants had a current smoking history (Table 3), with 72.42% of these having a pack year history of more than 10 years.

**TABLE 2:** Co-morbidities of the patients.

Condition	Number	%†
Diabetes mellitus type II	88	82.24
Hypertension	56	52.34
Ischaemic heart disease	7	6.54
Diabetes mellitus type I	4	3.74
Hypercholesterolaemia	4	3.74
Dyslipidaemia	4	3.74
Congestive cardiac failure	3	2.80
Cerebrovascular accident	2	1.87
Obesity	2	1.87
Asthma	2	1.87
Peripheral vascular disease	2	1.87
Hypertensive renal disease	2	1.87
Rheumatoid arthritis	2	1.87
Epilepsy	1	0.93
Gout	1	0.93
Actinic keratosis	1	0.93
Chronic kidney disease	1	0.93
Retinopathy	1	0.93
Hyperthyroidism	1	0.93
Pacemaker	1	0.93
Acute kidney injury	1	0.93
Gastro-oesophageal reflux disease	1	0.93
Benign prostatic hyperplasia	1	0.93
Non-alcoholic fatty liver disease	1	0.93
Previous coronary artery bypass graft	1	0.93
Previous amputation	1	0.93
Sarcoidosis	1	0.93
Polio	1	0.93
Critical limb ischaemia	1	0.93
TB	1	0.93
HIV	1	0.93

TB, tuberculosis; HIV, human immunodeficiency virus.

† , The total number of conditions is >100% because some patients presented with more than one comorbidity.

**TABLE 3:** History of smoking.

Smoking history	Number of participants	%
Current smoker	58	54.21
<10 pack years	16	27.58
>10 pack years	42	72.42
Previous smoker	4	3.74
Non-smoker	43	40.20
History of recreational drug use	2	1.87

### Amputation-related information

The most common indications for amputation were diabetic complications and peripheral vascular diseases, with trauma and malignancy being the least (Table 4). All participants had lower limb amputations, with most having amputations above the knee (52.34%) (Table 5). Only one participant had a double-limb amputation. The participants had a median hospital stay of 8 days (5–13) post-amputation. However, one participant had a longer hospital stay of 35 days. Post-operative complications were recorded in 19 participants, with sepsis (23.4%) being the most common complication. Other complications were delirium (2.47%), hematoma (1.23%) and renal failure (1.23%). A surgical revision was conducted on one participant.

**TABLE 4:** Indications for amputation.

Reason for amputation	Number of participants	%
Diabetic complications	56	52.34
Peripheral vascular disease	30	28.04
Infection	13	12.15
Sepsis of previous amputation	4	3.74
Trauma	3	2.80
Malignancy	1	0.93

**TABLE 5:** Types and levels of amputation performed.

Types and levels of amputation performed	Number of participants	%
Above-knee amputation	56	52.34
Below-knee amputation	33	30.84
Toectomy	9	8.4
Transmetatarsal amputation	7	6.54
Bilateral toectomy	1	6.54
Bilateral above-knee amputation	1	0.93
Bilateral below-knee amputation	1	0.93

## Discussion

The aim of this study was to describe the characteristics of patients who had undergone limb amputations at Groote Schuur Hospital during the years 2019 and 2020. The results of our study revealed that patients who presented at Groote Schuur Hospital for a LLA were generally women, over the age of 60 years, who had not completed school, and were a pensioner or not employed, with very low income and multiple co-morbidities.

The participants in this study had various co-morbidities with diabetes and hypertension the most prevalent. The patient profile in this study is consistent with that reported in a large South African study (*n* = 348) conducted at Grey’s Hospital. In that study, participants presented with co-morbidities including pre-existing cardiac disease, vascular disease, diabetes mellitus and hypertension (Khan et al. 2020). Interestingly, the most common indication for LLA in that study was also uncontrolled diabetes mellitus. Similarly, another retrospective review conducted in a Western Cape teaching hospital revealed that LLAs were commonly conducted on elderly females as a consequence of diabetic foot gangrene (Hagan et al. 2018). In contrast, a recent systematic review of studies investigating gender differences in diabetic-related amputations found that men were more likely to undergo amputations than women (Fan & Wu 2021). Our results could be an artefact of various barriers to access to specialised care, which affect women more than men in South Africa (Khan et al. 2020; Mutyambizi et al. 2019). Perhaps, the greater representation of women in the present and previous South African studies reflects a disempowered state of women in several areas in South Africa.

The disempowerment of women in South Africa is a common barrier for women wanting to seek primary healthcare. A previous South African-based study indicated that gender-based violence was a barrier in such a way that women were afraid to use chronic medication (e.g. for diabetes) out of fear of being stigmatised and abused by their partner (Mbali & Mthembu 2012). In addition, the study indicated gender roles as another important barrier, in that men, particularly in rural areas, are decision-makers and tend to dictate when women should seek and utilise healthcare. Similar findings are reported in a study conducted elsewhere in Africa (Azad et al. 2020). The barriers to accessing care faced by women suggest they are not receiving timely and effective care, thus increasing their likelihood of undergoing an amputation surgery to address complications resulting from uncontrolled diabetes conditions. In addition, the predominance of above-knee amputations and the data that one in five of the sample suffered from postoperative sepsis suggest that patients were presenting to hospital late in the progression of their disease. This highlights the need for programmes educating women and men about the importance of timely health care in managing diabetes and preventing associated amputations.

Our findings indicate that most participants who had undergone LLAs were pensioners and not involved in remunerative work. The current evidence indicates that people living in low-income areas have higher rates of LLAs than those living in affluent areas (Stevens et al. 2014). Low-income communities mostly comprise older people (>60 years) with multiple co-morbidities, which are often poorly managed (Abebe et al. 2020).

In our sample, low literacy levels, common in low-income areas, may have compounded vulnerability (Raghupathi & Raghupathi 2020). Over 80% of the sample had not completed school, suggesting associated low levels of health literacy. Indeed, over half the sample smoked and had multiple comorbidities, suggesting a lack of insight into one’s condition, how to live a healthy lifestyle, eat a suitable diet and a lack of knowledge regarding how and when to access healthcare (Bayati et al. 2018). There appears to be a need to target the poor quality of healthcare services in low socio-economic areas (Gordon, Booysen & Mbonigaba 2020) including offering education and empowerment programmes to improve health literacy. Poverty and its associated consequences may predispose people to a higher risk of amputation. Broad-based efforts should be made by the government and relevant stakeholders to combat poverty and improve levels of health literacy in low-income households to reduce the burden of poverty-related amputations and debilitating consequences.

LLAs are associated with physical impairment that has major mobility and psychological implications on an individual (Roșca et al. 2021). More specifically, above-knee amputations are associated with higher morbidity (Myers & Chauvin 2023), poor rehabilitation outcomes (Crane et al. 2021), and higher mortality (Hançerli & Doğan 2023) when compared with below-knee amputations. Given that over 50% of the participants in this study had above-knee amputations, it is plausible to infer that some may have challenges with returning to their highest level of function.

In fact, in a recent qualitative study, participants with LLAs expressed the need to live a functional life with dignity like everyone else (Limakatso 2022). They indicated that free movement and reduced barriers to access are important in facilitating improved functional independence and participation that is necessary for living a dignified and purposeful life. These findings indicate that undergoing LLA is a major life event with severe implications immediately post-amputation and in the long term. In consideration of this, it is imperative to implement pre-amputation education programmes designed to improve physical and psychological outcomes.

This study sheds some light on the sociodemographic and clinical profile of people undergoing LLAs at Groote Schuur Hospital. However, there are some limitations. In-depth information was not recorded in patient folders, limiting the range of data that could be extracted for this study. Therefore, some recommendations are based on relevant data reported elsewhere. In addition, several patient folders could not be found. Therefore, the study was underpowered because of a small sample size. In consideration of this, we conducted a post-hoc power analysis which showed 90% confidence in the results. While the results need to be interpreted with caution because of the sample size, our study does have important clinical implications and offers useful recommendations for improvement in healthcare.

### Recommendations

Uncontrolled diabetes was the primary indication for amputations at Groote Schuur Hospital. Therefore, we recommend that the early screening of comorbidities including diabetes be conducted to identify people who are at a high risk of undergoing an amputation and initiate timely treatment to prevent complications. In addition, health literacy projects focusing on healthy diet, adherence to medication and importance of staying active are recommended for preventing the complications arising from chronic diseases of lifestyle. Not only do health literacy projects have the potential to improve the patient’s quality of life, but they can also contribute towards reducing the burden of amputations and associated complications including depression, anxiety, loss of employment and disability.

## Conclusion

This study revealed that elderly people, most of whom are female, undergo amputations because of diabetic-related complications. These results are essential for informing healthcare professionals and patients about the risk factors for amputation, to use them as targets for prevention and management. Identifying these factors will enable healthcare professionals to identify patients who are at high risk of undergoing an amputation and prone to developing further complications as a result. This will enable healthcare providers to optimise healthcare and improve clinical outcomes of patients with limb amputations.
